# The Influence of Temperature, Storage Conditions, pH, and Ionic Strength on the Antioxidant Activity and Color Parameters of Rowan Berry Extracts

**DOI:** 10.3390/molecules26133786

**Published:** 2021-06-22

**Authors:** Elena Cristea, Aliona Ghendov-Mosanu, Antoanela Patras, Carmen Socaciu, Adela Pintea, Cristina Tudor, Rodica Sturza

**Affiliations:** 1 Department of Oenology and Chemistry, Food Technology, Faculty Food Technology, Technical University of Moldova, 9/9 Studentilor Street, MD-2045 Chisinau, Moldova; cristea.ele@gmail.com (E.C.); rodica.sturza@chim.utm.md (R.S.); 2 Department of Sciences, Faculty of Horticulture, “Ion Ionescu de la Brad” Iasi University of Life Sciences, 3 Mihail Sadoveanu Alley, 700490 Iasi, Romania; apatras@uaiasi.ro; 3 Department of Chemistry and Biochemistry, Faculty of Veterinary Medicine, University of Agricultural Sciences and Veterinary Medicine Cluj-Napoca, Manastur Street, 3–5, 400372 Cluj-Napoca, Romania; csocaciudac@gmail.com (C.S.); apintea@usamvcluj.ro (A.P.); cristina.tudor@usamvcluj.ro (C.T.)

**Keywords:** rowan berries, antioxidant activity, CIELab color parameters, polyphenols, carotenoids, bioaccessibility, organic acids, stability

## Abstract

Recent trends in the food industry combined with novel methods in agriculture could transform rowan into a valuable raw material with potential technological applications. Thus, the aim of this research was to investigate the content of bioactive compounds in its fruits and to assess the color and antioxidant stability of the extracts prepared from such fruits during various thermal treatments and at different pH and ionic strength values. Various spectrophotometric methods, HPLC, and capillary electrophoresis were used to quantify the concentrations of bioactive compounds—polyphenols, carotenoids, organic acids, and to assess antioxidant activity and color. The results show that rowan berries contain circa 1.34–1.47 g/100 g of polyphenols among which include catechin, epicatechin, ferulic acid methyl ester, procyanidin B1, etc.; ca 21.65 mg/100 g of carotenoids including zeaxanthin, *β*-cryptoxanthin, all-*trans*-*β*-carotene, and various organic acids such as malic, citric, and succinic, which result in a high antioxidant activity of 5.8 mmol TE/100 g. Results also showed that antioxidant activity exhibited high stability when the extract was subjected to various thermal treatments, pHs, and ionic strengths, while color was mainly impacted negatively when a temperature of 100 °C was employed. This data confirms the technological potential of this traditional, yet often overlooked species.

## 1. Introduction

*Sorbus aucuparia* L. is a *Rosaceae* family species interesting for its bright-colored yellow compounds which also possess functional properties. Some of its common names are mountain ash, rowan, keirn, cuirn, and witch wiggin tree. Native to the cooler regions of the northern hemisphere, it used to grow most often at high altitudes. Nowadays, this tree also serves for decorative purposes and can be seen in gardens and parks [1].

Its bright scarlet fruits are also known for their high content in potassium, calcium, and phosphorus, vitamin C, unsaturated fatty acids, and polyphenols, although discrepancies in concentrations due to growing region and climatic conditions have been reported. For the aforementioned reasons, many authors expressed support for future research on this non-traditional species, which can grow in regions with harsh climate and poor soil [2].

Due to recent trends in the food industry and the ever-growing desire for a clean label and “natural” declaration, combined with novel methods in agriculture such as permaculture [3,4], this species has become a great source of extracts with potential technological roles in food processes. Ongoing research shows that phenolic extracts from *Sorbus aucuparia* L. can protect oils from thermal and oxidative degradation during frying [5]. In another study, rapeseed oil was supplemented with phenolic extracts from rowan berries (*Sorbus aucuparia* L.) and Siberian apple tree (*Malus baccata*). The results of antiradical and antioxidant activity have demonstrated that natural extracts are more effective than BHT (butylated hydroxytoluene) and can be an alternative to synthetic antioxidants during frying and storing plant oils [5].

Nowadays, the increasing awareness of the benefits of fruits and vegetables for health, and the need for comfort due to an accelerated lifestyle have increased the demand for ready-to-eat products. In recent years, manufacturers have developed various foods, the purpose of which is to bring convenience to consumers. Food products are subjected to various technological treatments that may involve high temperatures, high pressure, microwaves, etc., and biological activity, as well as sensory characteristics such as color, which may change after such treatments [6]. Food composition and pH that can vary significantly, from very acidic with a pH of around 2–3 in products such as vinegar or lemon juice to mildly alkaline with a pH of about 7–8 in various ready-to-eat soups or cheeses, are also important factors for the stability of bioactive compounds and sensory properties. Furthermore, a large array of temperatures/time regimes are employed to ensure food safety [7].

The aim of this research was to investigate the content of bioactive compounds in rowan (*Sorbus aucuparia* L.) berries—polyphenols, organic acids, carotenoids, and their bioaccessibility. The influence of different thermal regimes, storage conditions, pH, and ionic strength on antioxidant activity and CIELab color parameters were investigated. This paper will provide some practical answers to researchers who study natural extracts which will subsequently be used either as food dyes or antioxidants, new product development specialists, and food technologists.

## 2. Results and Discussion

### 2.1. Polyphenol and Carotenoid Composition, Antioxidant Activity, and Carotenoid Bioaccessibility in Rowan Extracts

Table 1 presents the total polyphenol content tested using two different methods, as well for various classes of polyphenols, the antioxidant activity, the bioaccessibility of carotenoids, the carotenoids’ content, the concentration of various individual phenolics, carotenoids, and organic acids.

As the results show, rowan berry extract contains a high amount of polyphenols, i.e., between ca 1.34 g/100 g and 1.47 g/100 g depending on the testing method. A large proportion of these polyphenols are flavonoids.

As a result, antioxidant activity also exhibits high levels, which may be correlated with either polyphenol content or, also, a relatively high carotenoid content. With regards to polyphenol content, other authors report values between 4.27 and 8.19 g/kg fresh material, depending on the cultivar. The highest documented value from Mlcek et al. [2] of 8.19 ± 0.56 g/kg was reported for the Granatnaya variety. The cultivar, harvest year, soil, and climatic conditions are some of the factors that will affect the content of polyphenols. The documented antioxidant capacity varies between 6.58 g ascorbic acid/kg and 9.62 g ascorbic acid/kg, depending on the cultivar [2].

The documented flavonoid content varies between 3.11 g rutin/kg and 5.65 g rutin/kg. Fresh fruits were analyzed in the above-mentioned studies, however, considering that dry matter content in fresh berries is usually between 19% and 34% [8], the numbers obtained in the current study are consistent with what was previously reported. The content is similar to the ones of other species popular for their functional properties.

The main individual polyphenols identified in the extract were catechin, ferulic acid methyl ester, *p*-hydrozybenzoic acid, procyanidin B1, epicatechin, as well as gallic, protocatehuic, syringic, caffeic, ferulic, and chlorogenic acids. Trementzi et al. [9] analyzed the polyphenol composition in 24 different extracts and fractions obtained from *Sorbus domestica* fruits at five different stages of maturity. The authors identified 62 different polyphenols using the liquid chromatography coupled to diode array detection and electrospray ionization tandem mass spectrometry method (LC-DAD-MS (ESI+)) and determined that all maturity categories were rich in benzoic, phenylpropanoic, and cinnamoylquinic acids, as well as their derivatives. The mature fruit had a lower flavonoid content than the unripe fruit. All the fractions obtained in ethyl acetate, butanol, and water contained chlorogenic acid, and most of the flavonoids detected were flavonols (mainly quercetin), glycosides, and dimers. It should be noted that the types of flavonols determined and the antioxidant activity correlated with the maturity stage, and the extraction solvent [9].

Savikin et al. [10] report that caffeoylquinic acids such as neochlorogenic and chlorogenic acid were the most abundant compounds in *Sorbus aucuparia* L. berries regardless of the growing locality after analyzing fruits harvested in the Balkan peninsula. Zymone et al. [11] identified polyphenolic compounds in hydroalcoholic extracts from twenty varieties of rowan fruits. The authors found that neochlorogenic, chlorogenic, cryptochlorogenic acids, and di-caffeilquinic acid derivatives were detected in all rowan extracts. The significant variation of the flavone profile determined depended on the variety. Routine triplet, hyperoside, and isoquercitrin were detected in all sample extracts [11]. Bujor et al. [12] reported the presence of 15 phytochemical constituents in *Sorbus aucuparia* L. fruit extract among which included sorbitol, 2 flavonoid glycosides, and 12 organic and phenolic acids including malic, citric, neochlorogenic, and chlorogenic acids.

In the performed study, significant quantities, i.e., 333.70 mg/100 g of malic acid were found in rowan extracts, followed by citric (19.32 mg/100 g), and succinic (12.80 mg/100 g) acids. On the other hand, the levels of detected ascorbic acid were insignificant, i.e., 2.08 mg/100 g. Mrkonjić et al. [13] reported approximately 10 mg/100 g of ascorbic acid in rowan fruits.

Sergunova and Bocov [14] reported that the profile of organic acids in rowan fruits consists of the following acids: malic, citric, oxalic, succinic, tartaric, ascorbic, fumaric, quinic, and sorbic. Other authors have also shown that malic and citric acids reach significant amounts in the fruits of this species—2854.4 mg/kg and 1089.7 mg/kg respectively [15].

Rowan fruits are also rich in carotenoids 21.65 ± 0.27 mg/100 g. Zymone et al. [11] determined the total content of carotenoids in powders obtained by lyophilization from twenty varieties of rowan fruits and found that the values can vary a lot within a range starting with 39 µg/g DW (Kirsten Pink variety) and ending with 2659 µg/g DW (Dodong variety).

All-*trans*-*β*-carotene, *β*-cryptoxanthin, zeaxanthin, and *γ*-carotene were identified in the saponified extract following analysis by RP-HPLC. Carotenoids in rowan fruits have a high bioaccessibility of 15.3%. Bioaccessibility describes the amount of compound ingested, which is released from the food matrix during the digestion process and becomes available for intestinal absorption [16]. Aschoff et al. [17] demonstrated that the bioaccessibility of carotenoids in fresh and homogenized orange segments is 10.8 and 11.9%, respectively. Tudor et al. [18] reported that the bioaccessibility of carotenoids in sea buckthorn oil is 18.04%, and from oil-water emulsion—27.97%. In berries, carotenoids are associated with proteins: carotenes and lycopene form complexes with proteins embedded in chromoplasts, while lutein is localized in chloroplasts [19]. The formation of complexes between carotenoids and protein compounds, but also the crystalline state of carotenoids reduces their bioaccessibility [20]. However, processing operations, especially drying and grinding, reduce the particle size which favors the release of carotenoids, thus increasing their bioaccessibility. The positive effect of food processing on carotenoid bioaccessibility positively correlates with in vivo studies on carotenoid bioavailability, confirming that the consumption of processed plant foods improves carotenoid intake [21].

The bioaccessibility of carotenoids can be influenced by the isomers of the same compound, but which have a different behavior in the micelle. Carotenoid *trans* isomers are less likely to be incorporated into micelles than *cis* isomers because *trans* isomers tend to form aggregates, or because of their low solubility [22]. A higher micellarization was detected in the case of *cis*-lycopene and *cis*-*β*-carotene compared to *trans*-lycopene and *trans-β*-carotene [19]. Furthermore, in the plant matrix carotenoids can be incorporated differently into micelles and can compete at the micelle entry level [21]. According to Garrett et al. [23], the differential transfer of carotenoids in micelles depends on their hydrophilicity. Thus, lutein is micellarized to a greater extent than *α*-carotene and *β*-carotene, and xanthophylls (zeaxanthin, cryptoxanthin, rubixanthin) have higher bioaccessibility than carotenes, probably due to hydroxyl groups which help to increase their solubility in micellar structures [24].

Comparing the results of different studies on the in vitro bioaccessibility of carotenoids is very difficult. However, in vitro methods can help identify promising food matrices for carotenoid release, food processing conditions, storage, growth, and cultivation conditions, etc., and determine their potential impact on nutrient bioaccessibility.

### 2.2. Antioxidant Activity of the Mountain Ash Berry Extract in Different pH, Ionic Strength, and Temperature Conditions

**Figure 1:**
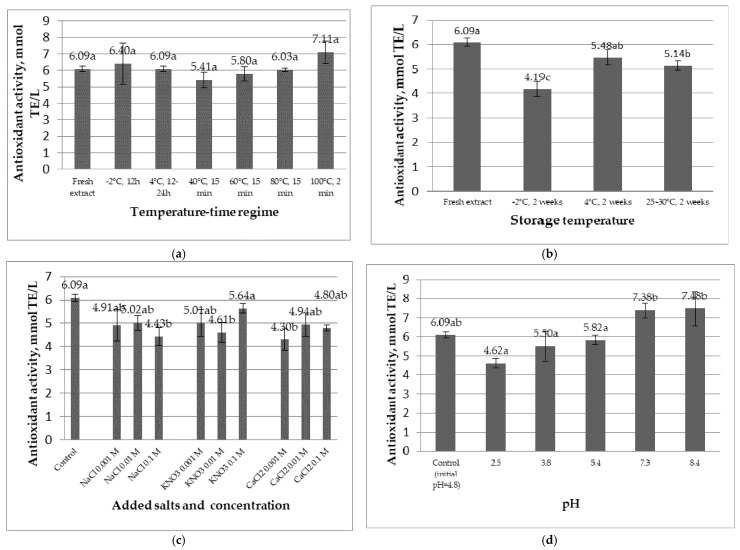
The antioxidant activity of the rowan extract after exposure to different thermal regimes (**a**), storage conditions (**b**), ionic strength (**c**), and pH (**d**) (results are presented as means ± standard deviations). Different letters ^(a–c)^ designate statistically different results (*p* < 0.05)).

Figure 1a presents the results for the antioxidant activity of rowan fruit extracts subjected to different thermal regimes. The antioxidant activity of the rowan fruit extract is stable at temperature as the statistical analysis showed no significant differences among different antioxidant activity values. The results from Figure 1b show a decrease in antioxidant activity when the extract was stored at t = −2 °C and t = 25–30 °C, with the highest impact exerted by the lowest temperature. The values of this parameter were reduced from 6.09 mmol TE/L to 4.19 mmol TE/L and 5.14 mmol TE/L, respectively. Hence, in the case of the rowan extract, the temperature of 4 °C was optimal for maintaining antioxidant activity.

Figure 1c presents the results for the change of antioxidant activity after the addition of sodium chloride, potassium nitrate, and calcium chloride, in different concentrations, to the rowan extract. The results show that all three salts produced significant differences in antioxidant activity. Sodium chloride caused a decrease in the investigated parameter when added at the concentration of 0.1 M. Potassium nitrate caused a decrease at the concentration of 0.01 M, although the value is not significantly different from the values determined for the other two concentrations. Calcium chloride caused a decrease in antioxidant activity at a concentration of 0.001 M, although there are no significant differences between that value and those determined for the other two studied concentrations.

Figure 1d shows the results of the antioxidant activity of the extract after the pH adjustment. In the case of the extract, the value of the antioxidant activity was not affected significantly by the pH change. Hence, this extract has proven to be stable in different pH environments. Nonetheless, a tendency towards higher values was observed for alkaline media which even results in a significant difference between antioxidant activity in acidic media and the one in alkaline media. Overall, media more acidic than the original pH of the rowan extract decreased antioxidant activity, while more alkaline media increased it. Similar results have been reported and thoroughly discussed for other extracts with similar composition, e.g. grape marc, aronia, and dog-rose berries [25,26].

### 2.3. CIELab Parameters of the Rowan Berry Extract in Different pH, Ionic Strength, Temperature Conditions

The color of plant extracts is a property of utmost importance. Table 2 presents the results for the color parameters of the extracts subjected to different temperatures for different time periods.

Only the treatment at 100 °C for 2 min produced a significant change in brightness compared to the control sample. Extracts exposed to −2 °C for 12 h; 4 °C for 12–24 h, 40 °C for 15 min, 60 °C for 15 min, and 80 °C for 15 min are lighter than the sample exposed at 100 °C for 2 min. The yellow/blue parameter dropped significantly in the extract exposed to −2 °C for 12 h, which suggests a degradation of the main yellow pigments, i.e., carotenoids. Hence, a study on the influence of sub-zero temperatures on the structure of model solutions containing carotenoids is recommended to fully understand the phenomenon.

For example, Valadon et al. [27] have identified *α*-, *β*-carotene, phytofluene, cryptoxanthin, monoepoxy-*α*-carotene, monoepoxy-*β*-carotene, aurochrome, and mutatochrome in *Sorbus aucuparia* berries from Surrey, United Kingdom, but also reported that their content varies significantly depending on the ripening stage.

It has been documented that even low temperature variations affect carotenoid concentration in citrus juice sacs culture systems [28], despite their high stability during thermal processing [29]. Other authors suggest that carotenoids such as *α*- and *β*-carotene, *β*-cryptoxanthin, lutein, and zeaxanthin are stable for at least 6 months at freezing temperatures (−20 °C and −70 °C) after the evaluation of their stability in working solutions. Only lycopene was stable for just 6 weeks [30].

The evolution of a* shows that red/green component will increase in time and demonstrate a shift of color to redder and browner tones, phenomenon which could be explained by oxidation reactions. Other authors who have researched the oxidation of carotenoids report the rate and explain the phenomenon as an attack on the double bond. The oxidative degradation will lead to a loss of color as the polyene chromophore is destroyed, although reactions on positions allylic to the polyene chain may also be involved [31]. The temperature will influence both the speed of the reaction as well as the availability of oxygen in the medium.

The chromaticity was higher in the extract exposed to 100 °C for 2 min. Additionally, the overall color difference value was the highest, i.e., 5.81. Thus, the extract exposed to 100 °C became slightly lighter in color, and its color saturation increased, whereas in those exposed to −2 °C the quality of the yellow shade decreased.

Table 3 presents the values of the CIELab parameters for specimens extract after storage for two weeks at different temperatures.

Storage at temperatures of −2 °C and 25–30 °C produced an increase in color luminosity (L*), while storage at t = 4 °C caused this parameter to decrease. However, these changes have not proven to be statistically significant. Moreover, the red/green component (a*) remained unchanged, and the yellow/blue component (b*) decreased significantly in the case of the extract stored at −2 °C, while increasing insignificantly in the other two cases.

The results for the overall color difference show that the best storage conditions are refrigeration at around 4 °C, whereas freezing and room temperature affected ΔE the most, resulting in values of 5.39 and 3.47, respectively.

Studies suggest that, first and foremost, oxygen availability influences the color exhibited by carotenoids, as well as its stability. Another important aspect is the physical state of the carotenoids themselves [32]. This might explain the higher stability at 4 °C. A future experiment should necessarily include the assessment of oxygen availability in stored tested solutions.

Table 4 presents the color parameters of the extract brought to different pH values. Acidic media generally had no significant effect on the color of the extract. Only in the case of pH = 2.5, the red/green (a*) parameter was decreased by 0.2, a change which was found to be significant. On the other hand, alkaline media namely 7.3 and 8.4 significantly changed the color parameters. Luminosity and yellow/blue parameter were affected the most and the color turned to a darker yellow. These changes resulted in increased chromaticity (C*) and significant color differences (ΔE) compared to controls. Such color modifications are caused by the degradations of carotenoids, but also polyphenols, widely reported by other authors [33,34].

Table 5 presents the color parameters after adding different salts to the extract. All salts produced significant changes in color parameters. The brightness was increased in the environment by two units in the case of KNO_3_ and NaCl addition and by three units in the case of addition of CaCl_2_, while the value of the red/green component decreased by about 0.4 units, resulting in a change of color to greener shades. The yellow/blue component was also modified, suggesting the degradation of yellow pigments. All these changes led to a significant decrease in the chromaticity or colorfulness of the extract. The increase in chroma is once again mainly due to the change in yellowness.

## 3. Materials and Methods

### 3.1. Materials

The *Sorbus aucuparia* L. berries were harvested from plantations from the Republic of Moldova. The ABTS reagent was provided by Alfa Aesar, the Folin–Ciocalteu reagent, formic acid, and acetronitrile were purchased from Merck (Darmstadt, Germany). (+)-catechin 98%, morin hydrate, ellagic acid (≥95%), benzoic acid, quercetin, caffeic acid, (+)-rutin trihydrate, syringic acid, ferulic acid, gallic acid (98%), protocatechuic acid, gentisic acid, parahydroxybenzoic acid, para-coumaric acid, quercetin (>95%), 3,5-dinitrobenzoic acid (99%), and cetyltrimethylammonium bromide were obtained from Sigma (Germany, Japan, China, India). D(-)-quinic acid (98%), sinapic acid (98%), and methyl 4-hydroxy-3-methoxycinnamate (99%) were purchased from Alfa Aesar (Kandel, Germany). Procyanidin B1, procyanidin B2, polydatin, hyperoside, carotenoid standards, *β*-carotene, lycopene, lutein, zeaxanthin, and *β*-cryptoxanthin were purchased from Extrasynthese (Genay, France). *Trans*-resveratrol was purchased from TCI Europe (Zwijndrecht, Belgium). All spectrophotometric measurements were performed on the Specord 200 Plus (Jena, Germany) spectrophotometer.

### 3.2. Methods

#### 3.2.1. Extraction

The harvested berries were dehydrated at a temperature up to 65 °C before extraction. After dehydration, the berries were milled to a fine powder and sieved. For the extraction, the sieved powder was stirred for 30 min in 50% vol. ethanol (1:10 ratio) solution at room temperature. The extract was then filtered and stored in dark glass bottles at 4 °C.

#### 3.2.2. Studies on the Ionic Strength

To study the effect of the ionic strength on the antioxidant activity and the color of the *Sorbus aucuparia* extract, three salts, namely sodium chloride, calcium chloride, and potassium nitrite, were added. The extracts containing added salts as well as the control were stored at t = 4 °C for 12 h, after which the antioxidant activity and the color parameters (CIELab) were measured.

#### 3.2.3. Studies on pH

The pH of the extract was adjusted to the following values: 2.5; 3.8; 5.4; 7.3, and 8.4 using appropriate buffers. For pH—2.5 the buffer was prepared: 2.1% citric acid and 20 mL NaOH 1 n per 100 mL buffer, adjusting the pH with HCl 0.1 n; for pH = 3.8: 0.75% glycocoll and 0.58% NaCl, adjusting the value with 0.1 n HCl; for buffer pH = 5.4: 2.1% citric acid and 20 mL NaOH 1 n per 100 mL buffer, adjusting the pH with HCl 0.1 n; for pH = 7.3: 19.2 mL KH_2_PO_4_ 0.9% and 80.8 mL Na_2_HPO_4_ 1.18% were mixed; for pH 8.4: 2 mL KH_2_PO_4_ 0.9% and 98 mL Na_2_HPO_4_ 1.18% were mixed and stored at t = 4 °C for 12 h. The antioxidant activity and the color parameters (CIELab) were measured for each pH as well as for the control sample which had an initial pH of 4.8.

#### 3.2.4. Antioxidant Activity by Reaction with ABTS Radical

The method described by Re at al. [35] which employs ABTS radical was employed to measure the antioxidant activity of the rowan berry extract. The results were expressed as mmol trolox equivalents per 100 g berry powder (mmol TE/100 g) from a calibration curve (0–2000 μmol/L) made using trolox as standard.

#### 3.2.5. Antioxidant Activity by Reaction with DPPH Radical

The antiradical DPPH activity of the rowan berry extract was measured following the method described by Brand-Williams et al. [36]. Results were expressed as mmol TE/100 g after the calibration curve (0–250 µmol/L) with trolox.

#### 3.2.6. Total Polyphenols and Flavonoids by Folin–Ciocalteu

The total polyphenols’ content was determined following the slightly modified method described by Ribereau-Gayon et al. [37] and the results were calculated from a calibration curve using gallic acid (0–500 mg/L) and expressed in equivalents of gallic acid per 100 g berry powder (mg GAE/100 g). A similar method which employs precipitation with formaldehyde and subsequent Folin–Ciocalteu reaction, described by Spranger et al. (2008) was used to determine the concentration of total flavonoids [38].

#### 3.2.7. Total Polyphenols by Absorbance at 280 nm

For comparison, the total polyphenol content was also determined using the method described by Ribereau-Gayon et al. [37] which employs absorption at 280 nm. The results were expressed as mg equivalent of gallic acid per 100 g berry powder (mg GAE/100 g) from a calibration curve (0–50 mg/L).

#### 3.2.8. Total Cinnamic Acids

The total cinnamic acid concentration was determined following the method described by Demir et al. [39]. The results were expressed as mg caffeic acid equivalents per 100 g berry powder (mg CAE/100 g) based on a calibration curve (0–50 mg/L) with standard of caffeic acid.

#### 3.2.9. Total Flavonols

The content of flavonols was determined following the method described by Demir et al. [39]. The results were expressed as mg quercetin equivalents per 100 g berry powder (mg QE/100 g) based on a calibration curve (0–50 mg/L, R^2^ = 0.9967) with standard of quercetin.

#### 3.2.10. Quantification of Organic Acids

The capillary electrophoresis method was used to determine the total content of organic acids. The optimal electrolyte was 10 mmol/L 3,5-dinitrobenzoic acid (DNB) at pH 3.6 containing 0.2 mmol/L cetyltrimethylammonium bromide as a flow inverter, according to Peres et al. [40]. The indirect detection of its UV absorption was made at 254 nm. The total content of organic acids was expressed in mg/100 g.

#### 3.2.11. Carotenoid Extraction and Determination by RP-HPLC

The methods described by Ghendov-Mosanu et al. [41,42] were followed to obtain an extract which was subsequently saponified and purified. Afterwards, the carotenoids were quantified by RP-HPLC [42,43]. The identification of carotenoids from rowan samples was carried out by the comparison of the UV–VIS spectra and the retention times of the sample peaks with those of the standard solutions (Table 6).

#### 3.2.12. Study on the Carotenoid Bioaccesibility

The static in vitro digestion model was applied to determine the bioaccessibility of carotenoids from plant powders, which consists of the gastric and intestinal phase, according to the method described by Tudor et al. [18].

#### 3.2.13. Analysis of Polyphenols by HPLC

The content of individual phenolics was analyzed using the Agilent 1100 Series HPLC (Santa Clara, CA, USA) following the method described by Cristea et al. [25].

The gradient was optimized using trifluoroacetic acid (TFA) as an eluent acidification of 1% CH_3_OH (A channel) and 50% CH_3_OH (B channel) acidified to 2.15 pH with TFA. The column system was composed of a pre-column SecurityGuard ULTRA Cartridges HPLC (Torrance, CA, USA) C18 for 4.6 mm ID coupled to a Kinetex 5 µm C18 100 Å 250 × 4.6 mm column manufactured by Phenomenex at 35 °C. The run time was 90 min, and the injection volume was 20 μL. The phases were A: H_2_O: CH_3_OH (99:1) and B: H_2_O: CH_3_OH (50:50), with a flow of 1.5 mL/min. The detection was carried out at 256, 280, 324, and 365 nm. The gradient of elution was 100% (A): for 10 min; 82% (A): 18% (B) for the next 10 min; 70% (A): 30% (B) for 10 min; 65% (A): 35% (B) for 6 min; 40% (A): 60% (B) for 15 min; 20% (A): 80% (B) for 5 min; 100% (B) for 15 min and 100% (A) for 10 min. The content of specific polyphenols was determined by comparison of retention times and peaks of the sample chromatogram with the ones from the chromatogram of a synthetic mixture containing the standards listed in Table 7 and Table S1.

#### 3.2.14. Color Parameters (CIELab)

A Specord 200 Plus (Jena, Germany) spectrophotometer and WinASPECT PLUS software (Jena, Germany) provided by the same company were used to assess color (CIELab) parameters as defined by the International Commission on Illumination/Commission Internationale de l’Eclairage. The transmittance of the samples was measured every nm, between 380 and 780 nm, in an optical glass cuvette with the path length of 1 mm, using distilled water as reference, D65 as illuminant and the observer placed at 10°. Three colorimetric coordinates, namely luminosity (L*), red/green component (a*), yellow/blue component (b*), and two derived magnitudes, namely chromaticity (C*) and hue (H*) are presented as results. The overall color difference (ΔE*) between the control and each tested extract was calculated, using the formula:

(1) $$\Delta E*=\sqrt{{\left(\Delta L*\right)}^{2}+{\left(\Delta a*\right)}^{2}+{\left(\Delta b*\right)}^{2}}$$

where ΔL*—difference of luminosity between the control and the sample with modified medium, Δa*—difference of red/green components between the control and the sample with modified medium, Δb*—difference of yellow/blue component between the control and the sample with modified medium [44].

#### 3.2.15. Statistical Analysis

The mean values and the standard deviations were calculated from 3 parallel experiments. One-way and two-way ANOVA and post-hoc Tukey test were used to distinguish between means and evaluate the results. The considered significance level was *p* ≤ 0.05. The calculations were performed using IBM SPSS Statistics 23 (Armonk, NY, USA).

## 4. Conclusions

The rowan berry contained circa 1.3–1.4 g/100 g of polyphenols depending on the testing method. The antioxidant activity thus also exhibits high concentrations, i.e., 5.8 mmol TE/100 g which can be attributed to polyphenols and carotenoids present in the plant material. The main phenolics identified were catechin and epicatechin, ferulic acid methyl ester, procyanidin B1, quercetin, and several phenolic acids including *p*-hydroxybenzoic, gallic, syringic, chlorogenic, ferulic, caffeic, and protocatechuic.

Rowan fruits are rich in carotenoids (21.65 ± 0.27 mg/100 g) with a high bioaccessibility of 15.3%. Their saponified extracts contain free all-*trans-β*-carotene, *β*-cryptoxanthin, zeaxanthin, and *γ*-carotene.

Rowan fruit extracts are also rich in organic acids. Malic acid is contained in significant amounts (333.70 mg/100 g), followed by citric (19.32 mg/100 g) and succinic (12.84 mg/100 g) acids. Ascorbic acid is contained at insignificant levels (2.08 mg/100 g).

The antioxidant activity was stable after the ethanolic extract was subjected to various thermal treatments, while the optimal tested storage temperature was 4 °C for both antioxidant activity and color. Nevertheless, the treatment at 100 °C for two minutes produced significant changes in the color parameters, which manifest as a loss of color vibrancy expressed as change in chromaticity.

Sodium chloride, potassium nitrate, and calcium chloride had a minor, but nonetheless significant effect on the antioxidant activity depending on the salt concentration. Their effect on the color was impactful regardless of the concentration, and these effects should be taken into account when the respective salts are used in foods.

The extract was stable in different pH environments; nevertheless a decreasing trend was observed for acidic values, while alkaline value increased slightly its antioxidant activity. pH values higher than 7 also produced important changes in color by shifting it towards redder tones. This data confirms the technological potential of this traditional yet overlooked and largely forgotten species used currently only as decoration.

## Figures and Tables

**Table 1:** Composition and antioxidant activity of the *Sorbus aucuparia* L. ethanolic extract (the results are expressed as means ± standard deviations of three experiments).

Compounds/Properties	Quantity
**Polyphenols**	
Total polyphenols (Folin-Ciocalteu), mg GAE/100 g	1468 ± 42
Total polyphenols (Abs280), mg GAE/100 g	1343 ± 176
Total flavonoids, mg GAE/100 g	525 ± 20
Cinnamic acids, mg CAE/100 g	383 ± 18
Flavonols, mg QE/100 g	242 ± 23
Catechin, mg/100 g	130.00 ± 10.00
Ferulic acid methyl ester, mg/100 g	13.80 ± 0.80
*p*-hydrozybenzoic acid, mg/100 g	10.70 ± 1.00
Procyanidin B1, mg/100 g	8.50 ± 0.80
Epicatechin, mg/100 g	7.40 ± 1.80
Gallic acid, mg/100 g	3.90 ± 0.10
Syringic acid, mg/100 g	3.70 ± 1.80
Polydatine, mg/100 g	3.70 ± 0.10
Chlorogenic acid, mg/100 g	2.80 ± 1.70
Ferulic acid, mg/100 g	2.50 ± 1.70
Caffeic acid, mg/100 g	2.10 ± 1.40
Protocatechuic acid, mg/100 g	1.80 ± 0.20
Quercetin, mg/100 g	1.20 ± 0.20
Sinapic acid, mg/100 g	0.70 ± 0.10
Vanillic acid, mg/100 g	0.40 ± 0.30
*p*-coumaric acid, mg/100 g	0.40 ± 0.10
*cis*-resveratrol, mg/100 g	0.20 ± 0.10
*trans*-resveratrol, mg/100 g	0.10 ± 0.10
Procyanidin B2, mg/100 g	Traces
Gentisic acid, mg/100 g	Traces
**Organic acids**	
Malic acid, mg/100 g	333.70 ± 11.2
Citric acid, mg/100 g	19.32 ± 1.41
Ascorbic acid, mg/100 g	2.08 ± 0.10
Succinic acid, mg/100 g	12.84 ± 0.52
Acetic acid, mg/100 g	7.58 ± 0.25
**Carotenoids**	
Total carotenoids, mg/100 g	21.65 ± 0.27
Carotenoid bioaccessibility, %	15.3 ± 1.89
Zeaxanthin, mg/100 g	1.11 ± 0.10
*β*-cryptoxanthin, mg/100 g	1.37 ± 0.08
*cis*-*β*-carotene, mg/100 g	0.15 ± 0.01
all-*trans*-*β*-carotene, mg/100 g	1.78 ± 0.15
*γ*-carotene, mg/100 g	0.10 ± 0.01
**Antioxidant activity**	
Antioxidant activity (ABTS), mmol TE/100 g	5.84 ± 0.34
Antioxidant activity (DPPH), μmol TE/100 g	1084 ± 16

ABTS = 2,20-azino-bis-3-ethylbenzthiazoline-6-sulphonic acid, DPPH = 2,2-diphenyl-1-picrylhydrazyl-hydrate.

**Table 2:** The CIELab color parameters of the *Sorbus aucuparia* extract submitted to different temperature/time regimes (results are presented as means ± standard deviations).

Temperature-Time Regime	L*	a*	b*	C*	H*	ΔE*
Fresh extract	94.41 ± 0.00 ^b,c^	−0.59 ± 0.00 ^a,b^	15.07 ± 0.00 ^b,c^	15.08 ± 0.00 ^b,c^	−4.92 ± 0.00 ^a^	-
−2 °C, 12 h	97.26 ± 0.10 ^c^	−0.92 ± 0.02 ^a,b^	11.38 ± 0.19 ^a^	11.42 ± 0.18 ^a^	−0.05 ± 7.75 ^a^	4.67 ± 0.21 ^a^
4 °C, 12–24 h	94.42 ± 0.00 ^b,c^	−3.09 ± 3.54 ^a^	15.07 ± 0.00 ^b,c^	15.58 ± 0.70 ^c^	−1.42 ± 4.95 ^a^	2.50 ± 3.54 ^a^
40 °C, 15 min	97.19 ± 0.04 ^c,d^	−0.96 ± 0.01 ^a,b^	12.32 ± 0.16 ^a,b^	12.36 ± 0.16 ^a,b^	1.62 ± 6.41 ^a^	3.93 ± 0.17 ^a^
60 °C, 15 min	96.83 ± 0.06 ^c,d^	−0.84 ± 0.06 ^a,b^	12.85 ± 0.17 ^a,b^	12.88 ± 0.17 ^a,b^	0.27 ± 0.88 ^a^	3.29 ± 0.19 ^a^
80 °C, 15 min	95.88 ± 0.21 ^b,c,d^	−0.74 ± 0.03 ^a,b^	14.23 ± 2.60 ^b,c^	14.25 ± 0.19 ^b,c^	−0.22 ± 0.94 ^a^	1.70 ± 2.61 ^a^
100 °C, 2 min	90.23 ± 2.54 ^a^	0.11 ± 0.44 ^b^	19.04 ± 2.60 ^c^	19.04 ± 2.60 ^d^	0.35 ± 2.42 ^a^	5.81 ± 3.66 ^a^

Different letters ^(a–d)^ designate statistically different results (*p* ≤ 0.05). L*—luminosity; a*—red/green component; b*—yellow/blue component; C*—chromaticity; H*—hue angle; ΔE*—overall difference of color.

**Table 3:** The CIELab color parameters of the *Sorbus aucuparia* extract during 2-week storage at different temperatures (results are presented as means ± standard deviations).

Storage Temperature and Time	L*	a*	b*	H*	C*	ΔE*
Fresh extract	94.41 ± 0.00 ^a,b^	−0.59 ± 0.00 ^a^	15.07 ± 0.00 ^b,c^	−4.92 ± 0.00 ^a^	15.08 ± 0.00 ^b,c^	-
−2 °C, 2 weeks	97.37 ± 0.10 ^b^	−0.71 ± 0.03 ^a^	10.75 ± 0.31 ^a^	−7.36 ± 14.42 ^a^	10.77 ± 0.31 ^a^	5.39 ± 0.31 ^a^
4 °C, 2 weeks	93.68 ± 1.44 ^a^	−0.51 ± 0.21 ^a^	15.65 ± 0.81 ^c^	−4.92 ± 0.00 ^a^	15.66 ± 0.80 ^c^	0.94 ± 1.67 ^b^
25–30 °C, 2 weeks	96.98 ± 0.05 ^a,b^	−0.58 ± 0.03 ^a^	12.74 ± 0.11 ^a,b^	0.50 ± 0.98 ^a^	12.75 ± 0.11 ^a,b^	3.47 ± 0.12 ^a^

Different letters ^(a–c)^ designate statistically different results (*p* ≤ 0.05). L*—luminosity; a*—red/green component; b*—yellow/blue component; C*—chromaticity; H*—hue angle; ΔE*—overall difference of color.

**Table 4:** CIELab parameters’ dependence on pH (results are expressed as means ± standard deviation).

pH	L*	a*	b*	C*	H*	ΔE*
Control 2.5	98.9 ± 0.0 ^a^	−0.5 ± 0.0 ^a^	4.7 ± 0.0 ^a^	4.7 ± 0.1 ^a^	−0.7 ± 1.7 ^a^	-
pH = 2.5	98.1 ± 0.4 ^a^	−0.3 ± 0.0 ^b^	5.1 ± 0.5 ^b^	5.1 ± 0.5 ^a^	5.0 ± 4.6 ^a^	0.92 ± 0.64 ^a^
Control 3.8	99.2 ± 0.0 ^a^	−0.5 ± 0.3 ^a^	4.1 ± 0.0 ^a^	4.2 ± 0.0 ^a^	0.6 ± 0.0 ^a^	-
pH = 3.8	99.1 ± 0.3 ^a^	−0.4 ± 0.0 ^a^	4.2 ± 0.3 ^a^	4.2 ± 0.3 ^a^	0.6 ± 0.6 ^a^	0.17 ± 0.52 ^a^
Control 5.4	98.5 ± 0.0 ^a^	−0.8 ± 0.0 ^a^	6.7 ± 0.2 ^a^	6.7 ± 0.2 ^a^	0.7 ± 0.2 ^a^	-
pH = 5.4	98.4 ± 0.1 ^a^	−0.7 ± 0.0 ^a^	6.4 ± 0.2 ^a^	6.5 ± 0.2 ^a^	5.0 ± 6.8 ^a^	0.51 ± 0.10 ^a^
Control 7.3	98.5 ± 0.0 ^a^	−0.8 ± 0.0 ^a^	6.7 ± 0.2 ^a^	6.7 ± 0.2 ^a^	0.7 ± 0.2 ^a^	-
pH = 7.3	97.9 ± 0.5 ^a^	−0.7 ± 0.0 ^a^	7.7 ± 0.6 ^b^	7.8 ± 0.6 ^b^	−6.9 ± 10.6 ^a^	1.22 ± 0.50 ^a^
Control 8.4	99.2 ± 0.0 ^a^	−0.5 ± 0.3 ^a^	4.1 ± 0.0 ^a^	4.2 ± 0.0 ^a^	0.6 ± 0.0 ^a^	-
pH = 8.4	97.4 ± 0.3 ^b^	−0.7 ± 0.1 ^b^	9.3 ± 0.3 ^b^	9.3 ± 0.3 ^b^	0.1 ± 1.3 ^a^	5.50 ± 9.31 ^a^

Different letters ^(a,b)^ designate statistically different results (*p* ≤ 0.05). L*—luminosity; a*—red/green component; b*—yellow/blue component; C*—chromaticity; H*—hue angle; ΔE*—overall difference of color.

**Table 5:** The CIELab color parameters after the addition of different concentrations of sodium chloride, potassium nitrate, and calcium chloride (results are presented as means ± standard deviations).

Salt and Concentration	L*	a*	b*	H*	C*	ΔE*
Control	94.41 ± 0.00 ^a^	−0.59 ± 0.00 ^a^	15.07 ± 0.00 ^a^	−4.92 ± 0.00 ^a^	15.08 ± 0.00 ^a^	-
NaCl 0.001 M	96.87 ± 0.01 ^c^	−0.95 ± 0.01 ^b^	12.81 ± 0.04 ^b^	−0.94 ± 0.44 ^a,b^	12.84 ± 0.04 ^b^	3.36 ± 0.04 ^a^
NaCl 0.01 M	96.87 ± 0.01 ^c^	−0.96 ± 0.00 ^b^	12.90 ± 0.02 ^b^	−0.75 ± 0.09 ^a,b^	12.93 ± 0.02 ^b^	3.30 ± 0.02 ^a^
NaCl 0.1 M	96.77 ± 0.06 ^c^	−0.93 ± 0.01 ^b^	12.83 ± 0.06 ^b^	−0.28 ± 0.08 ^a,b^	12.86 ± 0.06 ^b^	3.27 ± 0.09 ^a^
KNO_3_ 0.001 M	96.05 ± 0.01 ^b^	−0.86 ± 0.01 ^c,d^	13.32 ± 0.01 ^c^	0.12 ± 0.84 ^a,b^	13.35 ± 0.01 ^c^	3.89 ± 0.15 ^b^
KNO_3_ 0.01 M	95.79 ± 0.30 ^b^	−0.81 ± 0.03 ^c,d^	13.36 ± 0.16 ^c^	0.82 ± 1.62 ^a,b^	13.39 ± 0.16 ^c^	3.83 ± 0.12 ^b^
KNO_3_ 0.1 M	96.08 ± 0.20 ^b^	−0.82 ± 0.03 ^c,d^	13.24 ± 0.12 ^c^	1.68 ± 2.42 ^a,b^	13.27 ± 0.12 ^c^	3.91 ± 0.09 ^b^
CaCl_2_ 0.001 M	97.02 ± 0.04 ^c^	−0.81 ± 0.04 ^c,d^	12.19 ± 0.14 ^d^	6.40 ± 9.91 ^b^	12.21 ± 0.14 ^d^	2.41 ± 0.02 ^c^
CaCl_2_ 0.01 M	97.03 ± 0.11 ^c^	−0.80 ± 0.00 ^d^	12.29 ± 0.04 ^d^	2.44 ± 0.10 ^a,b^	12.32 ± 0.04 ^d^	2.21 ± 0.34 ^c^
CaCl_2_ 0.1 M	97.06 ± 0.03 ^c^	−0.81 ± 0.02 ^c,d^	12.21 ± 0.08 ^d^	1.93 ± 1.35 ^a,b^	12.24 ± 0.08 ^d^	2.49 ± 0.24 ^c^

Different letters ^(a–d)^ designate statistically different results (*p* ≤ 0.05). L*—luminosity; a*—red/green component; b*—yellow/blue component; C*—chromaticity; H*—hue angle; ΔE*—overall difference of color.

**Table 6:** Characteristics of carotenoid standards used in RP-HPLC analysis.

Compound	Max Absorption (nm)	Retention Time (min)
Zeaxanthin	426, 450, 476	10.253
*β*-Cryptoxanthin	428, 451, 476	35.002
*cis*-*β*-Carotene	424, 446, 472	69.374
all-*trans*-*β*-Carotene	421, 452, 478	74.513
*γ*-Carotene	434, 461, 488	82.639

**Table 7:** Polyphenols used as standards in HPLC analysis and their retention times.

Compound	Max Absorption (nm)	Retention Time (min)
Gallic acid	280	5.294
Protocatechuic acid	256	9.267
*p*-hydroxybenzoic acid	256	13.918
Gentisic acid	324	15.531
Procyanidin B1	280	16.704
*m*-hydroxybenzoic acid	280	17.989
Catechin	280	18.53
Vanillic acid	256	20.319
Caffeic acid	324	20.485
Chlorogenic acid	324	22.871
Procyanidin B2	280	23.433
Syringic acid	280	25.002
Epicatechin	280	26.836
*p*-coumaric acid	324	29.695
Ferulic acid	324	36.233
Polydatin	280	38.234
Sinapic acid	324	38.564
*trans*-resveratrol	324	49.333
*cis*-resveratrol	324	57.089
Ferulic acid methyl ester	365	57.754
Quercetin	256	65.278

## Data Availability

The data supporting the reported results can be found at the scientific library of the Technical University of Moldova and by contacting the authors.

## Supplementary Materials

The following are available online, Table S1: Identification and quantification of individual polyphenols in rowan berry extracts.

## Sample Availability

Samples of the rowan berries are available from the authors.
