# Supplementary material for: The Influence of Temperature, Storage Conditions, pH, and Ionic Strength on the Antioxidant Activity and Color Parameters of Rowan Berry Extracts
Source: Molecules. 2021 Jun 22;26(13):3786. doi: 10.3390/molecules26133786 (PMC8270275; doi:10.3390/molecules26133786)
Supplement: Supplementary file 1 [file molecules-26-03786-s001.zip › Chromatograms 2 P1.pdf]

This is a special file, named RPTHEAD.TXT, in the directory of a method
which allows you to customize the report header page.
It can be used to identify the laboratory which uses the method.

This file is printed on the first page with the report styles:

Header+Short, GLP+Short, GLP+Detail, Short+Spec, Detail+Spec, Full

```
                  XXXX   XXX
                 XX  XX   XX
                XX        XX       XXXXX   XXX XX
                XX        XX XXX  XX    X  XX X XX
                XX    X   XXX XX  XXXXXXX  XX X XX
                 XX  XX   XX  XX  XX       XX   XX
                  XXXX   XXX  XXX  XXXXX   XXX  XXX



      XXXXXX     X                 X        XX
     XX    X    XX                XX
     XX       XXXXX    XXXXX    XXXXX     XXX      XXXX    XX XXX
      XXXXX     XX          X     XX         XX     XX  XX   XXX XX
          XX    XX     XXXXXX     XX         XX     XX  XX   XX  XX
     X    XX    XX XX  X   XX     XX XX     XX     XX  XX   XX  XX
     XXXXXX      XXX   XXXXX X     XXX     XXXX     XXXX    XX  XX



                                                          X
          XX XXX    XXXXX   XX XXX     XXXX   XX XXX    XXXXX
           XXX XX  XX    X   XX  XX   XX  XX   XXX XX    XX
           XX      XXXXXXX   XX  XX   XX  XX   XX        XX
           XX      XX        XXXXX   XX  XX   XX        XX XX
          XXXX      XXXXX    XX        XXXX   XXXX        XXX
                            XXXX



          XXX                            XXX
           XX                            XX
           XX       XXXXX    XXXXX       XX    XXXXX   XX XXX
           XX XXX  XX    X       X    XXXXX   XX    X   XXX XX
           XXX XX  XXXXXXX  XXXXXX   XX  XX   XXXXXXX   XX
           XX  XX  XX       X   XX   XX  XX   XX        XX
          XXX  XXX  XXXXX   XXXXX X   XXXX X   XXXXX   XXXX



     X                                  XXX                X
    XX                                   XX               XX
   XXXXX    XXXXX   XXX XX   XX XXX      XX     XXXXX    XXXXX    XXXXX
    XX     XX    X  XX X XX   XX  XX     XX        X     XX     XX    X
    XX     XXXXXXX  XX X XX   XX  XX     XX    XXXXXX     XX     XXXXXXX
    XX XX  XX       XX   XX   XXXXX      XX    X   XX     XX XX  XX
     XXX    XXXXX   XXX  XXX  XX        XXXX   XXXXX X     XXX    XXXXX
                             XXXX
```

```
=====================================================================
Acq. Operator   : MariusN                        Seq. Line :   6
Acq. Instrument : Instrument 1                    Location : Vial 6
Injection Date  : 7/19/2015 11:29:07 AM                Inj :   1
                                                Inj Volume : 20.000 µl
Acq. Method     : C:\CHEM32\1\DATA\PATRAS 2015-07-19 03-45-27\ACFP1.M
Last changed    : 7/19/2015 12:35:07 AM by MariusN
Analysis Method : C:\CHEM32\1\METHODS\QUNATIF CF\QUALP1.M
Last changed    : 7/30/2015 7:56:07 PM by MariusN
                  (modified after loading)
=====================================================================
```

```
=====================================================================
Module                                 Type   Firmware rev.   Serial number
---------------------------------------|------|---------------|---------------
Binary Pump                            G1312A A.06.10 [005]   DE83103386
Sampler 2                              G1313A A.06.10 [006]   DE23921094
Column Comp. 3                         G1316A A.06.10 [004]   DE43651325
VWD 4                                  G1314A A.06.10 [004]   JP53500136

Software Revision: Rev. B.04.03-SP1 [87] Copyright © Agilent Technologies
=====================================================================
```

```
Instrument Conditions   :    At Start          At Stop
Column Temp. (left)     :      35.0              35.0   °C
Column Temp. (right)    :      35.0              35.0   °C
Pressure                :     162.2             163.3   bar
Flow                    :       1.500             1.500 ml/min

Solvent Description     :
PMP1, Solvent A         : 1% MeOH+1% TFA
PMP1, Solvent B         : MeOH:H2O 50:50+1% TFA
=====================================================================
                             Run Logbook
=====================================================================
30 Jul 15  07:57 PM
Logbook File:C:\CHEM32\...TRAS RPM\PATRAS 2015-07-19 03-45-27\006-0601.D\RUN.LOG
```

Module	# Event Message	Time	Date
Method	Method started: line# 6 at 6 inj# 1	11:27:20	07/19/15
Method	Instrument running sample Vial 6	11:27:24	07/19/15
G1314A	G1314A:JP53500136 - Detector: Prepare	11:27:32	07/19/15
G1314A	G1314A:JP53500136 - Detector: Idle	11:27:33	07/19/15
G1313A	G1313A:DE23921094 - Run	11:29:05	07/19/15
G1314A	G1314A:JP53500136 - Postrun	12:59:05	07/19/15
G1313A	G1313A:DE23921094 - Postrun	12:59:06	07/19/15
Method	Instrument run completed	12:59:15	07/19/15
Method	Saving Method ACFP1.M	12:59:19	07/19/15
Method	Saving Method RUN.M	12:59:28	07/19/15
CP Macro	Analyzing rawdata 006-0601.D	12:59:28	07/19/15
Method	Method completed	12:59:32	07/19/15

===

Data File C:\CHEM32\1\DATA\PATRAS RPM\PATRAS 2015-07-19 03-45-27\006-0601.D
Sample Name: scorus 2 P1

===

Acq. Operator : MariusN   Seq. Line : 6
Acq. Instrument : Instrument 1   Location : Vial 6
Injection Date : 7/19/2015 11:29:07 AM   Inj : 1
Inj Volume : 20.000 µl
Acq. Method : C:\CHEM32\1\DATA\PATRAS 2015-07-19 03-45-27\ACFP1.M
Last changed : 7/19/2015 12:35:07 AM by MariusN
Analysis Method : C:\CHEM32\1\METHODS\QUNATIF CF\QUALP1.M
Last changed : 7/30/2015 7:56:07 PM by MariusN
(modified after loading)

===

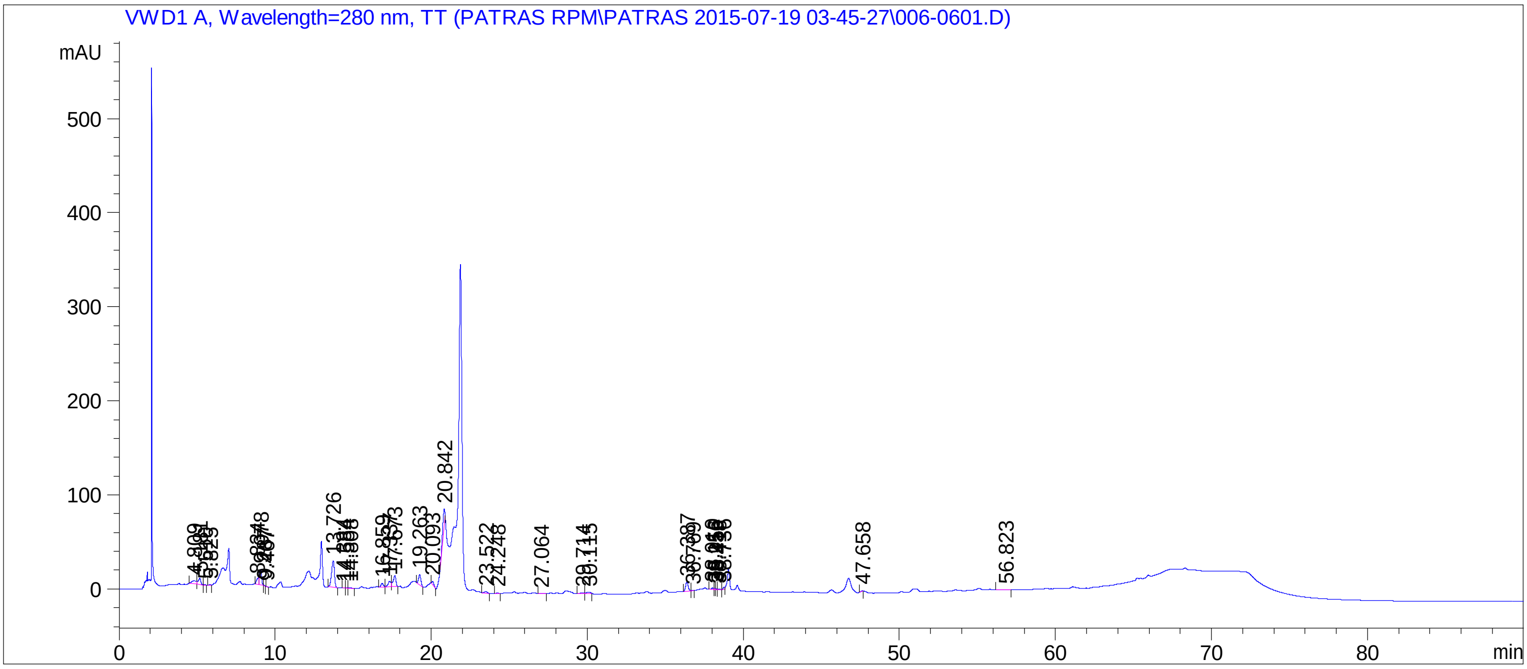

===

Area Percent Report

===

Sorted By : Signal
Multiplier : 1.0000
Dilution : 1.0000
Sample Amount: : 20.00000 [ng/ul] (not used in calc.)
Use Multiplier & Dilution Factor with ISTDs

Signal 1: VWD1 A, Wavelength=280 nm, TT

Peak #	RetTime [min]	Type	Width [min]	Area [mAU*s]	Height [mAU]	Area %
1	4.809	BV	0.2463	64.19932	3.59334	4.0352
2	5.181	VB	0.1636	79.50882	6.75122	4.9975
3	5.510	BB	0.0815	2.11434	4.03320e-1	0.1329
4	5.825	BB	0.0852	1.94666	3.58699e-1	0.1224
5	8.834	BV F	0.1933	59.93034	4.62801	3.7669
6	9.078	VB	0.1385	153.25182	16.34783	9.6326
7	9.287	BB	0.0837	2.97418	5.61199e-1	0.1869
8	9.467	BB	0.0866	2.16595	4.08844e-1	0.1361
9	13.726	BB	0.2048	364.84256	28.47472	22.9321
10	14.384	BB	0.1026	1.28589	2.08293e-1	0.0808

===

Acq. Operator : MariusN Seq. Line : 6
Acq. Instrument : Instrument 1 Location : Vial 6
Injection Date : 7/19/2015 11:29:07 AM Inj : 1
Inj Volume : 20.000 µl
Acq. Method : C:\CHEM32\1\DATA\PATRAS 2015-07-19 03-45-27\ACFP1.M
Last changed : 7/19/2015 12:35:07 AM by MariusN
Analysis Method : C:\CHEM32\1\METHODS\QUNATIF CF\QUALP1.M
Last changed : 7/30/2015 7:56:07 PM by MariusN
(modified after loading)

===

Peak #	RetTime [min]	Type	Width [min]	Area [mAU*s]	Height [mAU]	Area %
11	14.584	BV	0.0969	1.58876	2.42270e-1	0.0999
12	14.808	VB	0.1963	5.67945	4.10521e-1	0.3570
13	16.859	BV	0.1723	40.90634	3.62863	2.5712
14	17.337	VV	0.1932	77.38177	5.80975	4.8638
15	17.673	VB	0.1847	149.02614	11.83834	9.3670
16	19.263	BB	0.1610	109.65571	10.77279	6.8924
17	20.093	BB	0.1510	31.16294	3.38836	1.9587
18	20.842	BBA	0.2036	206.13841	16.87335	12.9568
19	23.522	BBA	0.1773	21.00604	1.81447	1.3203
20	24.248	BB	0.1625	4.10396	3.98044e-1	0.2580
21	27.064	BB	0.2080	3.42194	2.17873e-1	0.2151
22	29.714	BV	0.2445	13.95682	8.28840e-1	0.8773
23	30.115	VBA	0.2961	24.71799	1.34405	1.5536
24	36.387	BV	0.1747	110.34146	9.82468	6.9355
25	36.769	VB	0.0965	1.54812	2.46723e-1	0.0973
26	38.010	BV	0.1179	12.26510	1.48948	0.7709
27	38.156	VV	0.0886	4.96413	8.31781e-1	0.3120
28	38.256	VV B	0.0914	4.43016	7.27586e-1	0.2785
29	38.418	VB	0.1110	4.87524	6.27923e-1	0.3064
30	38.736	BV F	0.1345	18.87710	1.98045	1.1865
31	47.658	BBA	0.2037	4.75280	3.88812e-1	0.2987
32	56.823	BB	0.3769	7.95162	2.62370e-1	0.4998

Totals :				1590.97186	135.68258	

===

*** End of Report ***
