# Supplementary material for: The Influence of Temperature, Storage Conditions, pH, and Ionic Strength on the Antioxidant Activity and Color Parameters of Rowan Berry Extracts
Source: Molecules. 2021 Jun 22;26(13):3786. doi: 10.3390/molecules26133786 (PMC8270275; doi:10.3390/molecules26133786)
Supplement: Supplementary file 1 [file molecules-26-03786-s001.zip › Chromatograms 2 P2.pdf]

This is a special file, named RPTHEAD.TXT, in the directory of a method
which allows you to customize the report header page.
It can be used to identify the laboratory which uses the method.

This file is printed on the first page with the report styles:

Header+Short, GLP+Short, GLP+Detail, Short+Spec, Detail+Spec, Full

```
                  XXXX   XXX
                 XX  XX   XX
                XX        XX       XXXXX   XXX XX
                XX        XX XXX  XX    X  XX X XX
                XX    X   XXX XX  XXXXXXX  XX X XX
                 XX  XX   XX  XX  XX       XX   XX
                  XXXX   XXX  XXX  XXXXX   XXX  XXX



      XXXXXX     X                 X        XX
     XX    X    XX                XX
     XX       XXXXX    XXXXX    XXXXX     XXX      XXXX    XX XXX
      XXXXX    XX          X     XX        XX     XX  XX   XXX XX
          XX    XX     XXXXXX     XX        XX     XX  XX   XX  XX
     X    XX    XX XX  X   XX     XX XX     XX     XX  XX   XX  XX
     XXXXXX      XXX   XXXXX X     XXX     XXXX     XXXX    XX  XX



                                                          X
          XX XXX    XXXXX   XX XXX     XXXX   XX XXX    XXXXX
           XXX XX  XX    X   XX  XX   XX  XX   XXX XX    XX
           XX      XXXXXXX   XX  XX   XX  XX   XX        XX
           XX      XX        XXXXX    XX  XX   XX        XX XX
          XXXX      XXXXX    XX        XXXX   XXXX        XXX
                            XXXX



          XXX                          XXX
           XX                           XX
           XX       XXXXX    XXXXX       XX     XXXXX   XX XXX
           XX XXX  XX    X       X    XXXXX   XX    X   XXX XX
           XXX XX  XXXXXXX  XXXXXX   XX  XX   XXXXXXX   XX
           XX  XX  XX       X   XX   XX  XX   XX        XX
          XXX  XXX  XXXXX   XXXXX X   XXXX X   XXXXX   XXXX



     X                                  XXX                X
    XX                                   XX               XX
   XXXXX    XXXXX   XXX XX   XX XXX      XX     XXXXX    XXXXX    XXXXX
    XX      XX     X  XX X XX   XX  XX     XX         X     XX      XX    X
    XX      XXXXXXX  XX X XX   XX  XX     XX    XXXXXX     XX     XXXXXXX
    XX XX  XX       XX   XX   XXXXX      XX    X   XX     XX XX  XX
     XXX    XXXXX   XXX  XXX  XX        XXXX   XXXXX X     XXX    XXXXX
                             XXXX
```

```
=====================================================================
Acq. Operator   : MariusN                        Seq. Line :   6
Acq. Instrument : Instrument 1                    Location : Vial 6
Injection Date  : 7/22/2015 6:07:02 AM                 Inj :   1
                                               Inj Volume : 20.000 µl
Acq. Method     : C:\CHEM32\1\DATA\PATRAS RPM\PATRAS 2015-07-21 22-23-23\ACFP2.M
Last changed    : 7/17/2015 10:20:17 PM by MariusN
Analysis Method : C:\CHEM32\1\METHODS\QUNATIF CF\QUALP2.M
Last changed    : 7/30/2015 5:59:16 PM by MariusN
=====================================================================
Module                                 Type   Firmware rev.   Serial number
--------------------------------------|------|---------------|---------------
Binary Pump                            G1312A A.06.10 [005]   DE83103386
Sampler 2                              G1313A A.06.10 [006]   DE23921094
Column Comp. 3                         G1316A A.06.10 [004]   DE43651325
VWD 4                                  G1314A A.06.10 [004]   JP53500136

Software Revision: Rev. B.04.03-SP1 [87] Copyright © Agilent Technologies
=====================================================================

Instrument Conditions   :    At Start          At Stop
                          Not available

Solvent Description     :
PMP1, Solvent A         : 1% MeOH+1% TFA
PMP1, Solvent B         : MeOH:H2O 50:50+1% TFA
=====================================================================
                             Run Logbook
=====================================================================
30 Jul 15  08:21 PM
Logbook File:C:\CHEM32\...TRAS RPM\PATRAS 2015-07-21 22-23-23\006-0601.D\RUN.LOG

Module     # Event Message                                 Time     Date
-----------------------------------------------------------------------------
Method       Method started:  line# 6 at 6 inj# 1          06:05:14 07/22/15
Method       Instrument running sample Vial 6              06:05:19 07/22/15
G1314A       G1314A:JP53500136 - Detector: Prepare         06:05:27 07/22/15
G1314A       G1314A:JP53500136 - Detector: Idle            06:05:28 07/22/15
G1313A       G1313A:DE23921094 - Run                       06:06:59 07/22/15
G1314A       G1314A:JP53500136 - Postrun                   07:37:00 07/22/15
G1313A       G1313A:DE23921094 - Postrun                   07:37:06 07/22/15
Method       Instrument run completed                      07:37:11 07/22/15
Method       Saving Method ACFP2.M                         07:37:15 07/22/15
Method       Saving Method RUN.M                           07:37:24 07/22/15
CP Macro     Analyzing rawdata 006-0601.D                  07:37:24 07/22/15
Method       Method completed                              07:37:28 07/22/15
=====================================================================
```

Data File C:\CHEM32\1\DATA\PATRAS RPM\PATRAS 2015-07-21 22-23-23\006-0601.D
Sample Name: scorus 2 P2

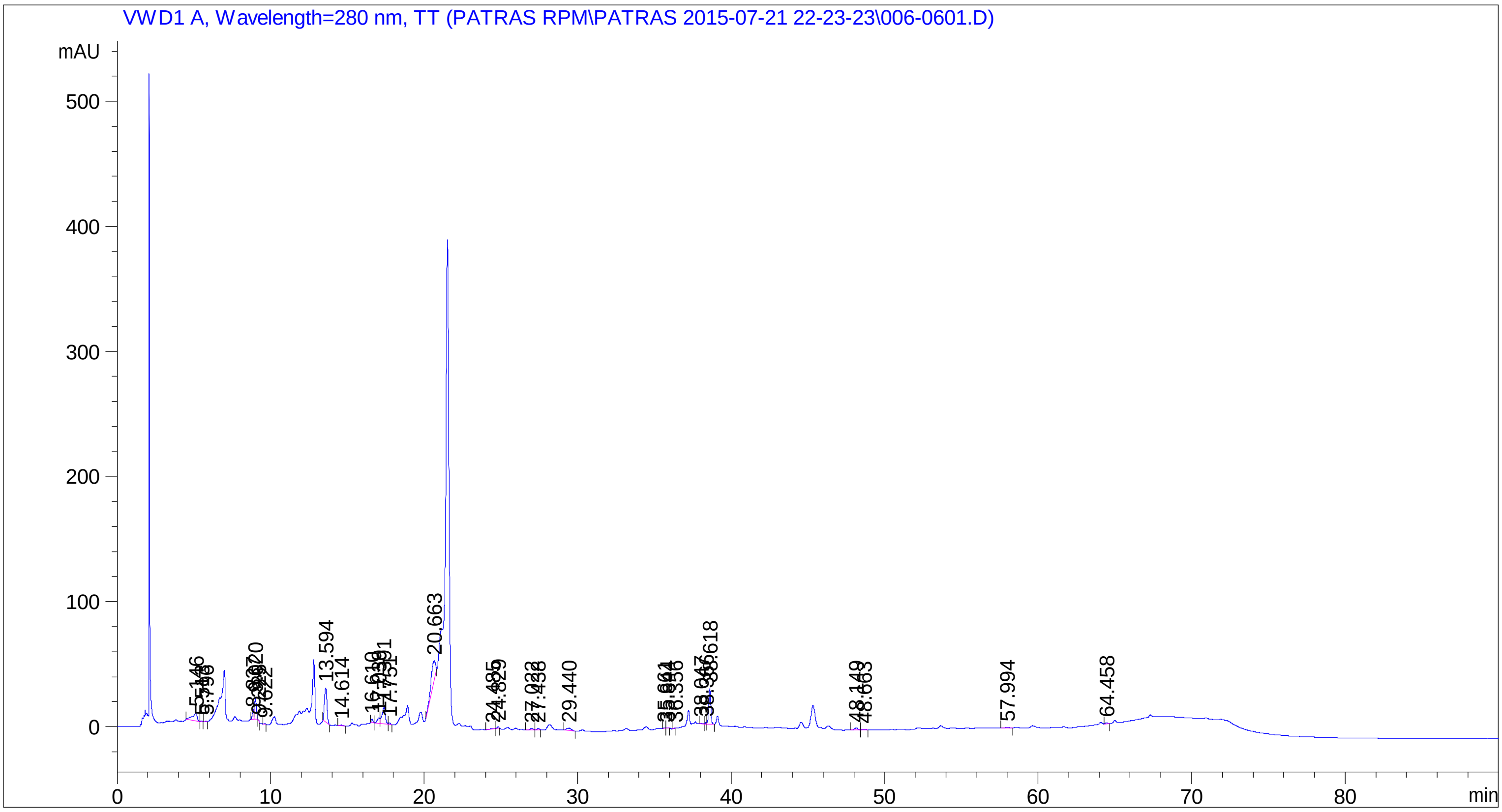

===

Area Percent Report

===

Sorted By : Signal
Calib. Data Modified : 7/30/2015 2:26:52 PM
Multiplier : 1.0000
Dilution : 1.0000
Sample Amount: : 20.00000 [ng/ul] (not used in calc.)
Use Multiplier & Dilution Factor with ISTDs

Signal 1: VWD1 A, Wavelength=280 nm, TT

Peak #	RetTime [min]	Type	Width [min]	Area [mAU*s]	Area %	Name
1	5.146	BB	0.2578	135.01190	7.9442	?
2	5.306		0.0000	0.00000	0.0000	ac galic
3	5.513	BB	0.0922	3.47269	0.2043	?
4	5.796	BB	0.0850	1.24259	0.0731	?
5	8.837	BV F	0.1204	44.74665	2.6329	?
6	9.020	VB	0.1458	156.71684	9.2214	?
7	9.225	BB B	0.0591	1.32781	0.0781	ac protocatehic
8	9.622	BBA	0.1077	5.08859	0.2994	?
9	13.594	BBA	0.1861	313.06104	18.4208	?
10	14.614	BB	0.1789	5.95440	0.3504	?
11	16.610	BB	0.1377	21.21263	1.2482	?
12	17.039	BV	0.1645	48.56015	2.8573	?
13	17.391	VV	0.2152	201.59526	11.8620	?
14	17.751	VBA	0.1340	13.48547	0.7935	?
15	20.663	BB	0.3502	346.57095	20.3926	?
16	24.485	BB	0.3316	7.20686	0.4241	?

Peak #	RetTime [min]	Type	Width [min]	Area [mAU*s]	Area %	Name
17	24.829	BBA	0.1281	6.95516	0.4092	?
18	27.022	BV	0.2182	12.95231	0.7621	?
19	27.438	VBA	0.2009	9.49084	0.5584	?
20	29.440	BB	0.3438	34.85197	2.0507	?
21	35.661	BV	0.1131	3.58420	0.2109	?
22	35.844	VBA	0.1400	2.26871	0.1335	?
23	36.356	BB	0.1088	1.86763	0.1099	?
24	38.047	BV	0.1325	5.03834	0.2965	?
25	38.366	VV	0.0864	7.68403	0.4521	?
26	38.618	VB	0.1370	258.47430	15.2089	?
27	48.149	BV	0.2606	25.23165	1.4847	?
28	48.663	VBA	0.2338	8.76487	0.5157	?
29	57.994	BB	0.3314	9.73971	0.5731	?
30	64.458	BB	0.1854	7.34019	0.4319	?
Totals :				1699.49775		

4 Warnings or Errors :

Warning : Calibration warnings (see calibration table listing)
Warning : Calibrated compound(s) not found
Warning : Invalid calibration curve, (ac protocatehic)
Warning : Amount limits exceeded

*** End of Report ***
