# Supplementary material for: The Influence of Temperature, Storage Conditions, pH, and Ionic Strength on the Antioxidant Activity and Color Parameters of Rowan Berry Extracts
Source: Molecules. 2021 Jun 22;26(13):3786. doi: 10.3390/molecules26133786 (PMC8270275; doi:10.3390/molecules26133786)
Supplement: Supplementary file 1 [file molecules-26-03786-s001.zip › Chromatograms P1.pdf]

This is a special file, named RPTHEAD.TXT, in the directory of a method
which allows you to customize the report header page.
It can be used to identify the laboratory which uses the method.

This file is printed on the first page with the report styles:

Header+Short, GLP+Short, GLP+Detail, Short+Spec, Detail+Spec, Full

```
                  XXXX   XXX
                 XX  XX   XX
                XX        XX       XXXXX   XXX XX
                XX        XX XXX  XX    X  XX X XX
                XX    X   XXX XX  XXXXXXX  XX X XX
                 XX  XX   XX  XX  XX       XX   XX
                  XXXX   XXX  XXX  XXXXX   XXX  XXX



      XXXXXX     X                 X        XX
     XX    X    XX                XX
     XX        XXXXX    XXXXX    XXXXX     XXX      XXXX    XX XXX
      XXXXX     XX          X     XX        XX     XX  XX   XXX XX
          XX    XX     XXXXXX     XX        XX     XX  XX   XX  XX
     X    XX    XX XX  X   XX     XX XX     XX     XX  XX   XX  XX
     XXXXXX      XXX   XXXXX X     XXX     XXXX     XXXX    XX  XX



                                                          X
          XX XXX    XXXXX   XX XXX     XXXX   XX XXX    XXXXX
           XXX XX  XX    X   XX  XX   XX  XX   XXX XX    XX
           XX      XXXXXXX   XX  XX   XX  XX   XX        XX
           XX      XX        XXXXX    XX  XX   XX        XX XX
          XXXX      XXXXX    XX        XXXX   XXXX        XXX
                            XXXX



          XXX                           XXX
           XX                            XX
           XX       XXXXX    XXXXX       XX    XXXXX   XX XXX
           XX XXX  XX    X       X    XXXXX   XX    X   XXX XX
           XXX XX  XXXXXXX  XXXXXX   XX  XX   XXXXXXX   XX
           XX  XX  XX       X   XX   XX  XX   XX        XX
          XXX  XXX  XXXXX   XXXXX X   XXXX X   XXXXX   XXXX



     X                                  XXX                X
    XX                                   XX               XX
   XXXXX    XXXXX   XXX XX   XX XXX      XX     XXXXX    XXXXX    XXXXX
    XX     XX    X  XX X XX   XX  XX     XX         X     XX     XX    X
    XX     XXXXXXX  XX X XX   XX  XX     XX    XXXXXX     XX     XXXXXXX
    XX XX  XX       XX   XX   XXXXX      XX    X   XX     XX XX   XX
     XXX    XXXXX   XXX  XXX  XX        XXXX   XXXXX X     XXX    XXXXX
                             XXXX
```

```
=====================================================================
Acq. Operator   : MariusN                          Seq. Line :  13
Acq. Instrument : Instrument 1                      Location : Vial 36
Injection Date  : 7/21/2015 1:51:18 PM                   Inj :   1
                                                 Inj Volume : 20.000 µl
Acq. Method     : C:\CHEM32\1\DATA\PATRAS RPM\PATRAS 2015-07-20 19-21-37\ACFP1.M
Last changed    : 7/20/2015 7:20:46 PM by MariusN
Analysis Method : C:\CHEM32\1\METHODS\QUNATIF CF\QUALP1.M
Last changed    : 7/30/2015 7:56:07 PM by MariusN
                  (modified after loading)
=====================================================================
```

Module	Type	Firmware rev.	Serial number
Binary Pump	G1312A	A.06.10 [005]	DE83103386
Sampler 2	G1313A	A.06.10 [006]	DE23921094
Column Comp. 3	G1316A	A.06.10 [004]	DE43651325
VWD 4	G1314A	A.06.10 [004]	JP53500136

Software Revision: Rev. B.04.03-SP1 [87] Copyright © Agilent Technologies

Instrument Conditions	At Start	At Stop	
Column Temp. (left)	35.0	35.0	°C
Column Temp. (right)	35.0	35.0	°C
Pressure	162.2	163.9	bar
Flow	1.500	1.500	ml/min

Solvent Description :
PMP1, Solvent A : 1% MeOH+1% TFA
PMP1, Solvent B : MeOH:H2O 50:50+1% TFA

Run Logbook

30 Jul 15 08:16 PM
Logbook File:C:\CHEM32\...TRAS RPM\PATRAS 2015-07-20 19-21-37\036-1301.D\RUN.LOG

Module	# Event Message	Time	Date
Method	Method started: line# 13 at 36 inj# 1	13:49:28	07/21/15
Method	Instrument running sample Vial 36	13:49:32	07/21/15
G1314A	G1314A:JP53500136 - Detector: Prepare	13:49:40	07/21/15
G1314A	G1314A:JP53500136 - Detector: Idle	13:49:42	07/21/15
G1313A	G1313A:DE23921094 - Run	13:51:16	07/21/15
G1314A	Starting analysis aborted via CAN	15:16:55	07/21/15
G1316A	Starting analysis aborted via CAN	15:16:55	07/21/15
Method	Instrument run completed	15:17:09	07/21/15
G1313A	Starting analysis aborted via CAN	15:17:11	07/21/15
Method	Saving Method ACFP1.M	15:17:17	07/21/15
Method	Saving Method RUN.M	15:17:25	07/21/15
CP Macro	Analyzing rawdata 036-1301.D	15:17:26	07/21/15
Method	Method stopped by user	15:17:29	07/21/15

===

Acq. Operator : MariusN Seq. Line : 13
Acq. Instrument : Instrument 1 Location : Vial 36
Injection Date : 7/21/2015 1:51:18 PM Inj : 1
Inj Volume : 20.000 µl
Acq. Method : C:\CHEM32\1\DATA\PATRAS RPM\PATRAS 2015-07-20 19-21-37\ACFP1.M
Last changed : 7/20/2015 7:20:46 PM by MariusN
Analysis Method : C:\CHEM32\1\METHODS\QUNATIF CF\QUALP1.M
Last changed : 7/30/2015 7:56:07 PM by MariusN
(modified after loading)

===

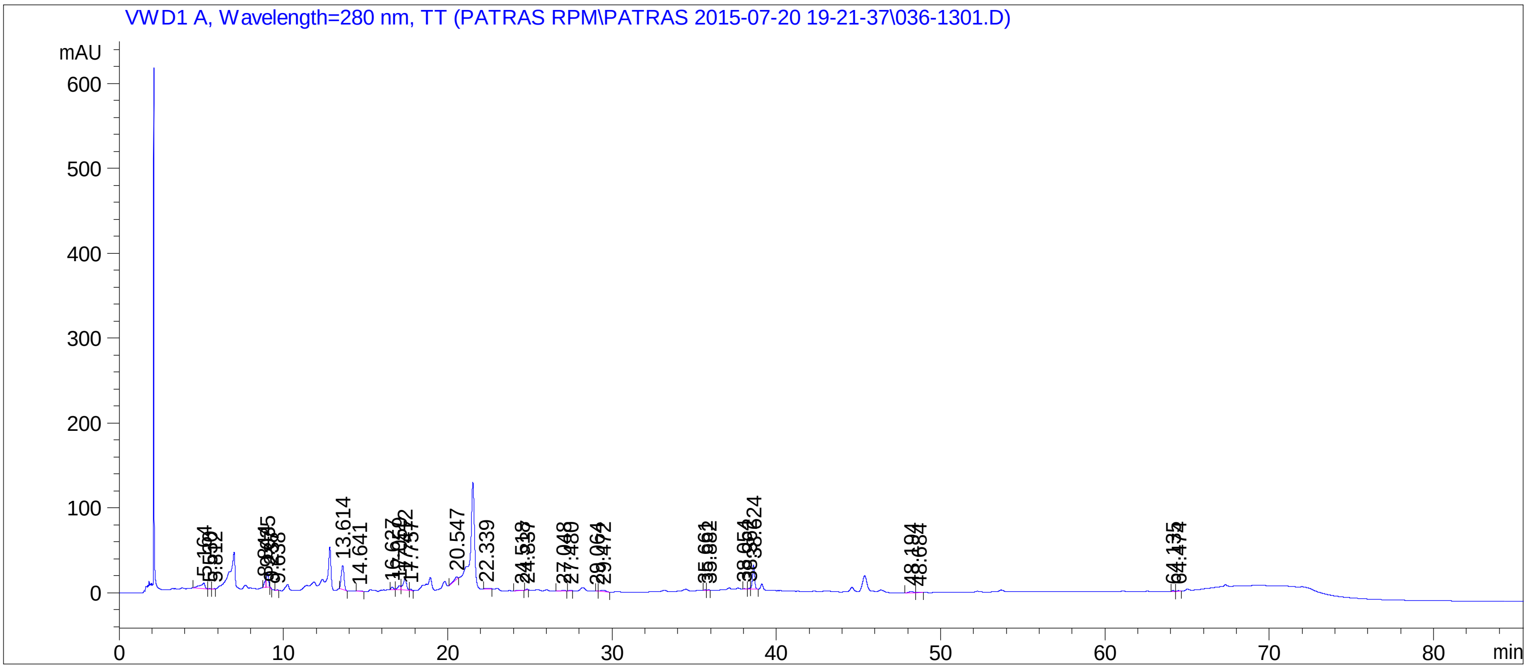

===

Area Percent Report

===

Sorted By : Signal
Multiplier : 1.0000
Dilution : 1.0000
Sample Amount: : 20.00000 [ng/ul] (not used in calc.)
Use Multiplier & Dilution Factor with ISTDs

Signal 1: VWD1 A, Wavelength=280 nm, TT

Peak #	RetTime [min]	Type	Width [min]	Area [mAU*s]	Height [mAU]	Area %
1	5.164	BB	0.2624	137.70805	6.84410	9.4669
2	5.530	BB	0.0899	3.50817	6.01333e-1	0.2412
3	5.812	BB	0.0792	1.12011	2.16737e-1	0.0770
4	8.844	BV F	0.1299	50.66176	5.62201	3.4828
5	9.035	VB	0.1453	159.46118	16.85275	10.9623
6	9.237	BB B	0.0614	1.69727	4.66496e-1	0.1167
7	9.638	BBA	0.1029	4.00403	6.46225e-1	0.2753
8	13.614	BB	0.1891	335.85852	28.65746	23.0890
9	14.641	BV	0.1891	6.47602	5.14571e-1	0.4452
10	16.627	BV	0.1586	29.96527	2.96800	2.0600

===

Acq. Operator : MariusN Seq. Line : 13
Acq. Instrument : Instrument 1 Location : Vial 36
Injection Date : 7/21/2015 1:51:18 PM Inj : 1
Inj Volume : 20.000 µl
Acq. Method : C:\CHEM32\1\DATA\PATRAS RPM\PATRAS 2015-07-20 19-21-37\ACFP1.M
Last changed : 7/20/2015 7:20:46 PM by MariusN
Analysis Method : C:\CHEM32\1\METHODS\QUNATIF CF\QUALP1.M
Last changed : 7/30/2015 7:56:07 PM by MariusN
(modified after loading)

===

Peak #	RetTime [min]	Type	Width [min]	Area [mAU*s]	Height [mAU]	Area %
11	17.059	VV	0.1848	64.79636	5.09257	4.4545
12	17.412	VV	0.2090	204.40417	14.17893	14.0520
13	17.757	VB	0.1285	12.70757	1.53675	0.8736
14	20.547	BB	0.2180	36.25429	2.44646	2.4923
15	22.339	BBA	0.2247	8.03422	5.30637e-1	0.5523
16	24.518	BB	0.3462	6.78607	2.54395e-1	0.4665
17	24.837	BBA	0.1213	5.92786	8.14754e-1	0.4075
18	27.048	BB	0.2310	14.31992	9.67323e-1	0.9844
19	27.480	BBA	0.1820	6.87719	6.40914e-1	0.4728
20	29.064	BV	0.1280	1.18367	1.24901e-1	0.0814
21	29.472	VB	0.3394	36.72852	1.74046	2.5249
22	35.661	BV	0.0951	2.38236	3.95171e-1	0.1638
23	35.882	VBA	0.1444	1.44429	1.66732e-1	0.0993
24	38.054	BV	0.1197	4.57726	5.37763e-1	0.3147
25	38.362	VV	0.0875	7.63030	1.32658	0.5246
26	38.624	VB	0.1350	258.60303	28.90206	17.7780
27	48.194	BV	0.2555	25.84833	1.57491	1.7770
28	48.684	VBA	0.2383	8.99701	5.97332e-1	0.6185
29	64.135	BB	0.1314	9.78850	1.23981	0.6729
30	64.474	BB	0.1743	6.87599	6.42710e-1	0.4727
Totals :				1454.62728	127.10085	

===

*** End of Report ***
