# Supplementary material for: The Influence of Temperature, Storage Conditions, pH, and Ionic Strength on the Antioxidant Activity and Color Parameters of Rowan Berry Extracts
Source: Molecules. 2021 Jun 22;26(13):3786. doi: 10.3390/molecules26133786 (PMC8270275; doi:10.3390/molecules26133786)
Supplement: Supplementary file 1 [file molecules-26-03786-s001.zip › Chromatograms P2.pdf]

```
This is a special file, named RPTHEAD.TXT, in the directory of a method
which allows you to customize the report header page.
It can be used to identify the laboratory which uses the method.

This file is printed on the first page with the report styles:

   Header+Short, GLP+Short, GLP+Detail, Short+Spec, Detail+Spec, Full




                  XXXX   XXX
                 XX  XX   XX
                XX        XX       XXXXX   XXX XX
                XX        XX XXX  XX    X  XX X XX
                XX    X   XXX XX  XXXXXXX  XX X XX
                 XX  XX   XX  XX  XX       XX   XX
                  XXXX   XXX  XXX  XXXXX   XXX  XXX




      XXXXXX     X                 X        XX
     XX    X    XX                XX
     XX        XXXXX    XXXXX    XXXXX     XXX      XXXX    XX XXX
      XXXXX     XX          X     XX        XX     XX  XX   XXX XX
          XX    XX     XXXXXX     XX        XX     XX  XX   XX  XX
     X    XX    XX XX  X   XX     XX XX     XX     XX  XX   XX  XX
     XXXXXX      XXX   XXXXX X     XXX     XXXX     XXXX    XX  XX




                                                          X
          XX XXX    XXXXX   XX XXX     XXXX   XX XXX    XXXXX
           XXX XX  XX    X   XX  XX   XX  XX   XXX XX    XX
           XX      XXXXXXX   XX  XX   XX  XX   XX        XX
           XX      XX        XXXXX    XX  XX   XX        XX XX
          XXXX      XXXXX    XX        XXXX   XXXX        XXX
                            XXXX




          XXX                           XXX
           XX                            XX
           XX       XXXXX    XXXXX       XX     XXXXX   XX XXX
           XX XXX  XX    X       X    XXXXX   XX    X   XXX XX
           XXX XX  XXXXXXX  XXXXXX   XX  XX   XXXXXXX   XX
           XX  XX  XX       X   XX   XX  XX   XX        XX
          XXX  XXX  XXXXX   XXXXX X   XXXX X   XXXXX   XXXX



     X                                  XXX                X
    XX                                   XX               XX
   XXXXX    XXXXX   XXX XX   XX XXX      XX     XXXXX    XXXXX    XXXXX
    XX     XX    X  XX X XX   XX  XX     XX         X     XX     XX    X
    XX     XXXXXXX  XX X XX   XX  XX     XX    XXXXXX     XX     XXXXXXX
    XX XX  XX       XX   XX   XXXXX      XX    X   XX     XX XX  XX
     XXX    XXXXX   XXX  XXX  XX        XXXX   XXXXX X     XXX    XXXXX
                             XXXX
```

===

Acq. Operator : MariusN Seq. Line : 36
Acq. Instrument : Instrument 1 Location : Vial 36
Injection Date : 7/24/2015 4:15:45 AM Inj : 1
Inj Volume : 20.000 µl
Acq. Method : C:\CHEM32\1\DATA\PATRAS RPM\PATRAS 2015-07-21 22-23-23\ACFP2.M
Last changed : 7/17/2015 10:20:17 PM by MariusN
Analysis Method : C:\CHEM32\1\METHODS\QUNATIF CF\QUALP2.M
Last changed : 7/30/2015 5:59:16 PM by MariusN

===

Module	Type	Firmware rev.	Serial number
Binary Pump	G1312A	A.06.10 [005]	DE83103386
Sampler 2	G1313A	A.06.10 [006]	DE23921094
Column Comp. 3	G1316A	A.06.10 [004]	DE43651325
VWD 4	G1314A	A.06.10 [004]	JP53500136

Software Revision: Rev. B.04.03-SP1 [87] Copyright © Agilent Technologies

===

Instrument Conditions	At Start	At Stop	
Column Temp. (left)	35.0	35.0	°C
Column Temp. (right)	35.0	35.0	°C
Pressure	161.4	162.7	bar
Flow	1.500	1.500	ml/min

Solvent Description :
PMP1, Solvent A : 1% MeOH+1% TFA
PMP1, Solvent B : MeOH:H2O 50:50+1% TFA

===

Run Logbook

===

30 Jul 15 08:38 PM
Logbook File:C:\CHEM32\...TRAS RPM\PATRAS 2015-07-21 22-23-23\036-3601.D\RUN.LOG

Module	# Event Message	Time	Date
Method	Method started: line# 36 at 36 inj# 1	04:13:53	07/24/15
Method	Instrument running sample Vial 36	04:13:58	07/24/15
G1314A	G1314A:JP53500136 - Detector: Prepare	04:14:08	07/24/15
G1314A	G1314A:JP53500136 - Detector: Idle	04:14:08	07/24/15
G1314A	G1314A:JP53500136 - Run	04:15:42	07/24/15
G1314A	G1314A:JP53500136 - Postrun	05:45:43	07/24/15
G1313A	G1313A:DE23921094 - Postrun	05:45:48	07/24/15
Method	Instrument run completed	05:45:53	07/24/15
Method	Saving Method ACFP2.M	05:45:57	07/24/15
Method	Saving Method RUN.M	05:46:06	07/24/15
CP Macro	Analyzing rawdata 036-3601.D	05:46:06	07/24/15
Method	Method completed	05:46:10	07/24/15

===

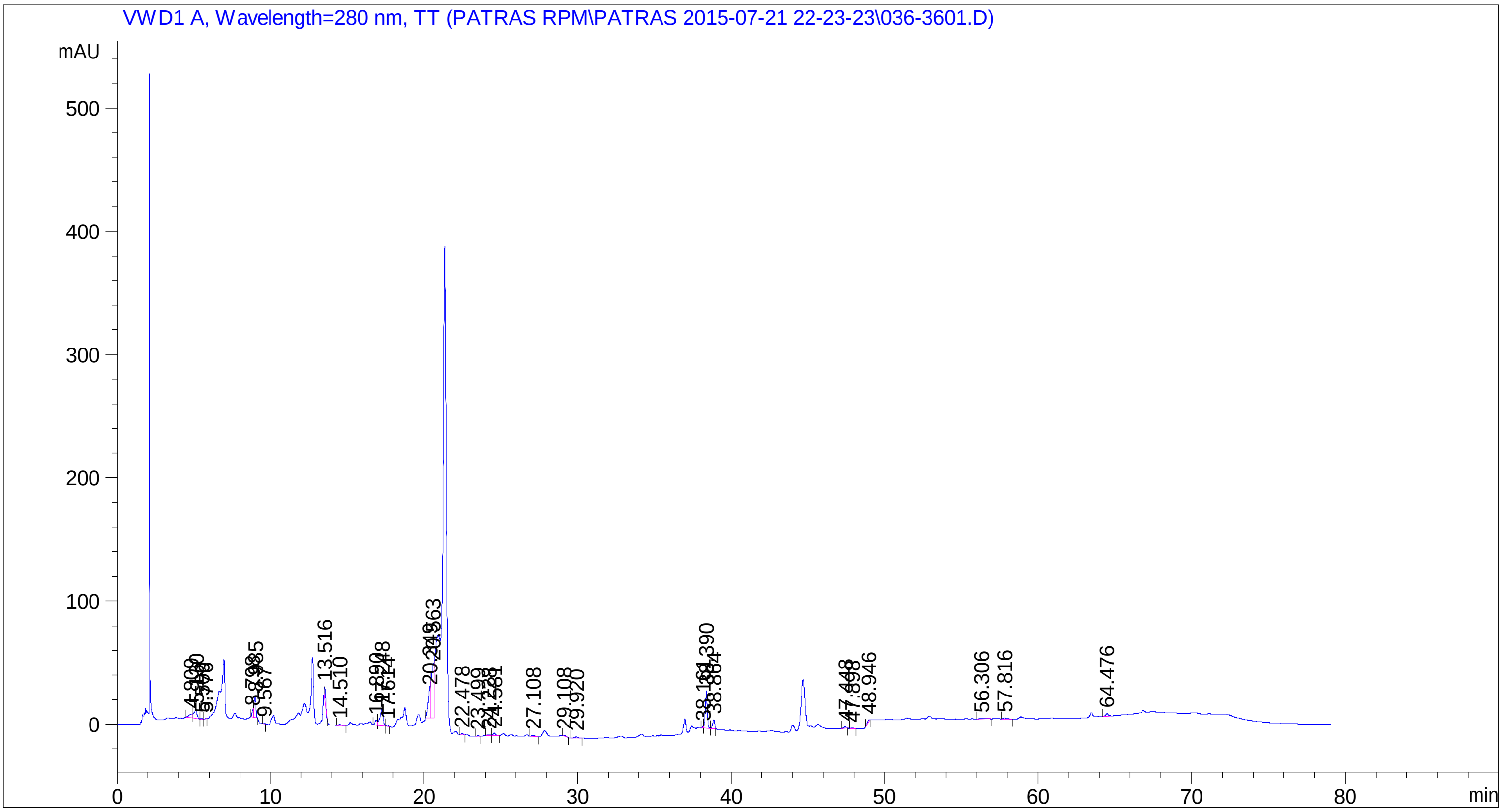

===

Area Percent Report

===

Sorted By : Signal
Calib. Data Modified : 7/30/2015 2:26:52 PM
Multiplier : 1.0000
Dilution : 1.0000
Sample Amount: : 20.00000 [ng/ul] (not used in calc.)
Use Multiplier & Dilution Factor with ISTDs

Signal 1: VWD1 A, Wavelength=280 nm, TT

Peak #	RetTime [min]	Type	Width [min]	Area [mAU*s]	Area %	Name
1	4.809	BV F	0.2602	49.97486	2.5387	?
2	5.130	VB	0.1877	91.22134	4.6339	?
3	5.306		0.0000	0.00000	0.0000	ac galic
4	5.500	BB	0.0870	3.01955	0.1534	?
5	5.776	BB	0.0938	1.81752	0.0923	?
6	8.798	BV F	0.1205	37.67969	1.9141	?
7	8.985	VB	0.1375	146.22241	7.4279	ac protocatehic
8	9.567	BBA	0.1127	6.70085	0.3404	?
9	13.516	BB	0.1083	69.27859	3.5193	?
10	14.510	BB	0.1893	9.70570	0.4930	?
11	16.890	BV	0.1555	41.71957	2.1193	?
12	17.248	VV	0.2089	196.54671	9.9843	?
13	17.614	VB	0.1177	10.45246	0.5310	?
14	20.349	BV F	0.1814	286.24451	14.5409	?
15	20.563	VV F	0.1873	535.41937	27.1986	?
16	22.478	BB	0.1591	8.03990	0.4084	?

Peak #	RetTime [min]	Type	Width [min]	Area [mAU*s]	Area %	Name
17	23.499	VB	0.1585	3.85125	0.1956	?
18	24.228	BV	0.2148	4.11010	0.2088	?
19	24.581	VBA	0.1710	19.21250	0.9760	?
20	27.108	BB	0.2326	13.85343	0.7037	?
21	29.108	BB	0.2445	9.00984	0.4577	?
22	29.920	BBA	0.3048	16.36063	0.8311	?
23	38.161	BV F	0.0799	7.97574	0.4052	?
24	38.390	VV	0.1306	264.41437	13.4319	?
25	38.864	VBA	0.1336	60.49092	3.0729	?
26	47.448	BBA	0.2166	14.70116	0.7468	?
27	47.898	BB	0.2072	5.18811	0.2635	?
28	48.946	BB F	0.1536	14.43082	0.7331	?
29	56.306	BB	0.3418	6.24014	0.3170	?
30	57.816	BBA	0.2564	10.62773	0.5399	?
31	64.476	BBA	0.1904	24.04264	1.2213	?

Totals : 1968.55240

4 Warnings or Errors :

Warning : Calibration warnings (see calibration table listing)
Warning : Calibrated compound(s) not found
Warning : Invalid calibration curve, (ac protocatehic)
Warning : Amount limits exceeded

===

*** End of Report ***
