# Supplementary material for: The Influence of Temperature, Storage Conditions, pH, and Ionic Strength on the Antioxidant Activity and Color Parameters of Rowan Berry Extracts
Source: Molecules. 2021 Jun 22;26(13):3786. doi: 10.3390/molecules26133786 (PMC8270275; doi:10.3390/molecules26133786)
Supplement: Supplementary file 1 [file molecules-26-03786-s001.zip › Table S1.pdf]

Table S1. HPLC chromatograms of standard compounds and all tested extracts.

Peak	Retention Time (min)	Area, mAU·s
1	4.809	64.19932
2	5.181	79.50882
3	5.510	2.11434
4	5.825	1.94666
5	8.834	59.93034
6	9.078	153.25182
7	9.287	2.97418
8	9.467	2.16595
9	13.726	364.84256
10	14.384	1.28589
11	14.584	1.58876
12	14.808	5.67945
13	16.859	40.90634
14	17.337	77.38177
15	17.673	149.02614
16	19.263	109.65571
17	20.093	31.16294
18	20.842	206.13841
19	23.522	21.00604
20	24.248	4.10396
21	27.064	3.42194
22	29.714	13.95682
23	30.115	24.71799
24	36.387	110.34146
25	36.769	1.54812
26	38.010	12.26510
27	38.156	4.96413
28	38.256	4.43016
29	38.418	4.87524
30	38.736	18.87710
31	47.658	4.75280
32	56.823	7.95162
